# An “out of the box” approach for prevention of ketoacidosis in youth with poorly controlled type 1 diabetes: combined use of insulin pump and long-acting insulin

**DOI:** 10.1007/s00592-024-02264-7

**Published:** 2024-05-18

**Authors:** Galia Barash, Liat Lerman, Tal Ben-Ari, Shirly Abiri, Zohar Landau, Michal Ben Ami, Avivit Brener, Yael Lebenthal, Orit Pinhas-Hamiel, Kineret Mazor-Aronovitch, Alon Haim, Yonatan Yeshayahu, Liat De Vries, Marianna Rachmiel

**Affiliations:** 1 Pediatric Endocrinology and Diabetes Institute, Shamir (Assaf Harofeh) Medical Center, Tzrifin, Beer Ya’akov, Israel; 2 Faculty of Medicine, Tel Aviv University, Tel Aviv, Israel; 3 The Jesse Z. and Sara Lea Shafer Institute for Endocrinology and Diabetes, National Center for Childhood Diabetes, Schneider Children’s Medical Center of Israel, Petah Tikva, Israel; 4 Pediatric Endocrine and Diabetes Unit, Edith Wolfson Medical Center, Holon, Israel; 5 National Juvenile Diabetes Center, Maccabi Health Care Services, Ra’anana, Israel; 6 Faculty of Health Sciences, Ben-Gurion University of the Negev, Beer‑Sheva, Israel; 7 Pediatric Endocrine and Diabetes Unit, The Edmond and Lily Safra Children’s Hospital, Chaim Sheba Medical Center, Ramat‑Gan, Israel; 8 Institute for Pediatric Endocrinology, Diabetes and Metabolism, Dana-Dwek Children’s Hospital, Tel Aviv Sourasky Medical Center, Tel Aviv, Israel; 9 Pediatric Endocrine and Diabetes Unit, Soroka Medical Center, Beer Sheba, Israel; 10 Pediatric Endocrine and Diabetes Unit, Assuta Medical Center, Ashdod, Israel

**Keywords:** Adolescents, Diabetic ketoacidosis, Glycemic control, Type 1 diabetes, Poorly controlled

## Abstract

**Background:**

Poorly controlled adolescents living with type 1 diabetes (T1D) and pump failure of insulin delivery leading to diabetic ketoacidosis (DKA) are still challenging in the western world.

**Aim:**

To investigate the effect of a combination modality of long-acting insulin for basal coverage and a pump for boluses, on the incidence of DKA and glycemic parameters in pediatric and young adults with poorly controlled T1D.

**Methods:**

This multicenter, observational retrospective study included 55 patients (age range 3–25 years, 52.7% males) who were treated with the combination modality for a median of 18 months [(IQR)12,47], as part of their clinical care. Data were retrieved at initiation of the combined modality, after 6 months, and at last visit.

**Results:**

Cohort’s median age at combination modality initiation was 14.5 years [IQR12.4,17.3], and its median HbA1c level was 9.2% [IQR 8.2,10.2]. The main reasons for combination modality initiation were: (a) concern about sustained hyperglycemia on current management in 41.8%, (b) previous DKA episodes in 30.8%, and (c) refusal to wear a pump continuously in 14.6%. The percent of patients experiencing DKA who used the modality till end decreased from 25.4 to 8.8%. The frequency of DKA events per patient month decreased after 6 months from 0.073 (min 0, max 0.5) to 0.020 (min 0, max 0.5), *p* = 0.01, and at end to 0.016 (min 0, max 0.25), *p* = 0.007.

**Conclusions:**

The combination modality of once-daily long-acting insulin and pump for boluses is safe, feasible, and effective in preventing DKA among poorly controlled young people living with T1D, unable or un-willing to use advanced closed pumps.

## Introduction

Managing type 1 diabetes (T1D) in pediatric as well as adults remains a significant challenge for patients and caregivers. Only a minority of people living with T1D meet widely accepted glycemic goals, though it should be noted that studies with various advanced hybrid closed-loop (AHCL) using automated insulin delivery demonstrate improvement in glycemic outcomes (1). Despite the advances in technology and introduction of new insulins, up to 10% of people living with diabetes continue to experience acute episodes of diabetic ketoacidosis (DKA) after diagnosis [2]. Management of T1D includes multiple daily blood glucose assessments by continuous glucose monitoring (CGM), or by 4–6 self-monitoring finger prick tests, and insulin administration by subcutaneous multiple daily injections (MDI), or by continuous subcutaneous insulin administration by an insulin pump, which is considered the most physiologic method of insulin replacement [3]. Pump use affords the ability to administer accurate doses of insulin, adjust doses to activity, use extended boluses for food containing more fat and proteins, and improve glycemic control and quality of life [4–6]. Recent reports have suggested that the AHCL system provides even more benefits [1, 7, 8]. However, in many countries they are still not available to most people living with diabetes due to economic status, and systems availability. Some limitations of the use of non-automated pumps have been identified from real-life data, following their extensive use among people living with T1D, including; refusal to wear the pumps constantly (mainly among adolescents, females, and those with worse glycemic control) [9], frequent local skin irritation and allergic reactions at the insertion site [5], and increased incidence of diabetic ketoacidosis (DKA) [10, 11]. DKA is the most distressing complication of pump therapy, reported to be associated with lower socioeconomic status [16], and mostly occurring due to insulin infusion set blockage, infusion site problems, and users delay in detection of those issues (e.g., misjudging prolonged hyperglycemia, prolonged suspension of insulin basal rate for physical activities) [12, 13]. When basal insulin infusion rates are interrupted among those treated with pumps and not recognized in time, the subcutaneous reserves of short-acting insulin are insufficient to prevent the metabolic processes that lead to ketogenesis. [14] This complication has been shown to be possibly fatal [15], and its occurrence is reported in association with low socioeconomic status. [16].

A few case reports have suggested using daily subcutaneous long-acting insulin instead of the basal pump rate, in addition to boluses by the pump, to prevent DKA [17–20]. Accordingly, pediatric endocrinologists in Israel implemented this proposed combination of pump for meal boluses and corrections of hyperglycemia and long-acting insulin for basal coverage as part of clinical care among poorly controlled T1D adolescents, needing a pump for boluses, during the last decade. The aim of this multicenter study was to describe the Israeli national real-life experience of this combination modality, in prevention of DKA episodes and improving glycemic control parameters among pediatric and young adults living with T1D.

## Methods

### Study design

This multicenter retrospective nationwide study was conducted to include all children, youth, and young adults whose T1D was managed with the combination modality that had been initiated by their medical teams. The modality consisted of either full or partial replacement of their basal insulin by once-daily long-acting insulin (Degludec, Detemir, or Glargine) administered subcutaneously, and a low basal pump rate of at least 0.1 unit per hour over a 24-h period (to prevent crystallization of insulin in the pump catheter). The boluses for meals and correction of hyperglycemia were delivered by the insulin pump with rapid-acting insulin (Lispro or Aspart). The protocol was not uniform, but rather tailored for each patient by the physician. The reported data were retrieved from the medical files, recorded glucometer results, description of the CGM devices, and pump features acquired from Dexcom Clarity, CareLink, and Tidepool softwares. Retrieval from those systems was carried out over the two-week periods preceding the initiation of the combination modality (baseline), 6 months since baseline, and at the last follow-up visit.

### Study population

All participants diagnosed as having T1D according to ADA [21], who were within the age range of 3–25 years and treated with this combination modality in the participating medical centers, were eligible for study enrollment. The medical teams who used this regimen sporadically in Israel were affiliated with the Shamir (Assaf Harofeh) Medical Center, Schneider Children’s Medical Center of Israel, the Edith Wolfson Medical Center, the Edmond and Lily Safra Children’s Hospital, the Chaim Sheba Medical Center, Dana-Dwek Children’s Hospital of the Tel Aviv Sourasky Medical Center, the Soroka Medical Center, and the Assuta Medical Center. Exclusion criteria were lack of essential data in the medical charts and databases and treatment with the modality for less than 3 months.

### Data retrieval

The information retrieved from the medical files and databases included: demographic characteristics (age, sex), household (1 or 2 parents at home), Israeli socioeconomic position (SEP) cluster (range 1–10) and index (range − 2.79 to 2.59) by home address, based upon the Israel Central Bureau of Statistics Socio-Economic Level of the Population 2015 [22], clinical data of coexisting morbidities, including autoimmune diseases (thyroid and celiac) and attention deficit hyperactivity disorder (ADHD), diabetes duration, mode of insulin therapy, reason for starting the combination modality as reported by the pediatric endocrinologist, and anthropometric data (weight, height, body mass index [BMI]). [23] The BMI standard deviation score (SDS) and height SDS were calculated by CDC 2000 growth charts. The Tanner stage of puberty [24] was defined according to breast development in females and testicular volume in males as measured with Prader beads by board-certificated experts in pediatric endocrinology. Specific diabetes management information, including data on DKA, HbA1c levels, and the occurrence of severe hypoglycemic episodes (loss of consciousness and need for assistance) during the 6 months prior to each time point, was collected. The information retrieved from the two-week ambulatory glucose and insulin profile reports included: mean and SDS of glucose levels, mean total daily dose of insulin per kg (TDDi), and the mean percent of basal insulin. Also, the reports included, when CGMS were used, the percent time spent in various glycemic ranges: time-in-range (TIR) (70–180 mg/dL; 3.9–10 mmol/L), hypoglycemic range (< 70 mg/dL; < 3.8 mmol/L), and hyperglycemic range (> 180 mg/dL; > 10 mmol/L).

### Outcome measures

Primary outcome measures were: (a) the difference in the percentage of DKA episodes among all participants, and the mean number of episodes of DKA per patient month at the end of study, and after 6 months, compared to baseline, and (b) the difference in HbA1c and the mean glucose level at those time points. Secondary outcome measure included the characteristics of patients chosen for that type of management.

### Statistical analysis

Continuous variables were examined for normal distribution and described by the median and interquartile range (IQR) or the mean and SDS, or range. Categorical variables were described by frequency and percentage. The Wilcoxon test and McNemar test were used to compare baseline and six-month periods and baseline and the end of follow-up. The chi-square test was used to compare categorical variables between patients who were still on the combination treatment at study closure and those who stopped the combination treatment, and the Mann–Whitney test was used to compare continuous variables between them. All of the tests were two-sided. Statistical significance was defined as *P* < 0.05. The statistical analyses were performed by NCSS 2021 Statistical Software (NCSS, LLC. Kaysville, Utah, USA).

## Results

### Description of baseline characteristics of the study population

The study cohort comprised of 55 individuals (52.7% males) who started the combination modality treatment at a median age of 14.5 years [IQR 12.4, 17.3] and were followed for a median duration of 18 months [IQR 12, 47]. Table 1 displays their baseline sociodemographic, anthropometric, clinical, and glycemic control characteristics. Their median age at T1D diagnosis was 8.3 years [IQR 6.5, 10.7], and their median HbA1c level was 9.2% [IQR 8.2, 10.2], 77 mmol/mol [IQR 66, 88]. The study cohort derived from a medium socioeconomic background, with a median SEP cluster of 6 [IQR 4,7], and a median SEP index of 0.29 [IQR 0.3,1.17]. Twenty-one (38%) of the study population had comorbid medical conditions: 7 (13%) had another autoimmune disease (celiac and Hashimoto), 11 (20%) had attention deficit hyperactivity disorder (ADHD), and 3 (5.5%) had other illnesses (asthma and epilepsy). At baseline, 35 (64%) of the adolescents were using a pump exclusively, and 21 (38%) were using CGM for glucose monitoring, and their percentages of time spent in the different ranges are reported in the first column of Table 2.

**Table 1:** Demographic, anthropometric, clinical, and glycemic control characteristics of the study population at baseline (*n* = 55)

Characteristics				Percent or median and IQR
Demographic	Sex (Male)			52.7%
	Age (years)			14.5 (12.4, 17.3)
	Age at T1D diagnosis (years)			8.33 (6.5, 10.66)
	SEP cluster ^			6 (4, 7)
	SEP index^			0.29 (− 0.30,1.17)
	Household (2 parents at home)			76.9%
	Siblings			3 (1, 4)
Anthropometric	BMI SDS			0.5 (− 0.2, 1.07)
	Height SDS			− 0.58 (− 1.12, 1.23)
	Pubertal stage		Tanner 1	24%
			Tanner 2	5%
			Tanner 3	14%
			Tanner 4	11%
			Tanner 5	56%
Diabetes-related clinical and glycemic control	MDI			36%
	Medtronic pump (G640,Veo)			46%
	Omnipod pump			18%
	Use of CGMS			38%
	HbA1c (%)			9.2 (8.2, 10.2)
	HbA1c mmol/mol			77 ( 66, 88)
	% time spent in range*	70–180 mg/dL		41 (35 66.25)
		< 180 mg/dL		56 (42.375 67.5)
		< 70 mg/dL		3.5 (1, 11)
	Insulin total daily dose/kg/d			0.85 (0.67, 0.98)
	% of basal insulin			48 (37.75, 57.75)

^The socioeconomic position (SEP) by home address was analyzed based upon the Israel Central Bureau of Statistics’ Characterization and Classification of Statistical Areas within Municipalities and Local Councils by the Socio-Economic Level of the Population according to home address [21]

CGMS—continuous glucose monitoring system

*Data available for only 21/55 of the study population

**Table 2:** Glycemic control and physical examination parameters during the periods of follow-up (*n* = 55)

Characteristics	Baseline	6 months	*P* value*	Last follow-up	*P* value*
Use of CGMS	38%	40%	0.25	51%	< 0.001
HbA1c (%)	9.20 (8.2, 10.2)	9.35 (7.9, 10.2)	0.20	9.17 (8, 10.6)	0.26
HbA1c (mmol/mol)	77) 66, 88)	79 (63, 88)		77 (64, 92)	
Mean glucose	221 (181, 266)	205 (176, 240)	**0.03**	200 (180, 251)	0.27
SD of mean glucose levels	89 (74.5,110.2)	80 (60,101.5)	**0.02**	82 (67.5, 102)	0.91
% time spent in 70–180 mg/dL	41((35,66)	44 (31,66)	0.20	41 (31,55)	0.97
% time spent in > 180 mg/dL	56 (42,68)	54 (28,67	0.03	55 (30,67)	0.64
% time spent in < 70 mg/dL	3 (1,11)	2 (1,10)	0.86	4 (1,13)	0.50
Insulin total daily dose U/kg/d	0.85 (0.67,0.99)	0.93 (0.75,1.11)	0.11	0.91(0.75,1.17)	0.37
% of patients with DKA episodes	25.4%	7.2%	**0.013**	20%	0.45
DKA episodes per patient^a^	0.51 ± 0.93	0.085 ± 0.28	**0.002**	0.28 ± 0.67	0.09
DKA events per patient months^a^	0.07 ± 0.15	0.02 ± 0.08	**0.01**	0.016 ± 0.04	0.01
BMI SDS	0.50 (− 1.3, 1.1)	0.50 (-0.01, 1.33)	**0.05**	0.59 (0.12, 1.09)	0.71
Height SDS	− 0.58 (− 1.12, 0.23)	− 0.69 (− 1.79, 0.01)	**0.04**	− 0.59(− 1.3, 0.16)	0.75

^*^P value of difference from baseline, ^a^mean and (minimum and maximum), Bold indicates significant

The main reason for switching to the combination modality was the physician’s impression of incompetence of safe usage of the pump (*n* = 23, 41.8%) by the caregivers, including fear from future DKA. Other reasons were: family fear to rely on technology (*n* = 3, 5.5%), an event of DKA while being treated with a pump (*n* = 17, 30.8%), and refusal to be constantly connected to the pump (*n* = 8, 14.6%). Less common causes included body image concerns, athletes, dancers, and among those who used very high doses of insulin in a patch pump (Omnipod), which require frequent pod changes (*n* = 4, 7.3%).

### Analysis of rate of DKA episodes among patients and per patient month

Fourteen of the study population (25.5%) had a reported episode of DKA during the 6 months prior to the combination modality, and this sub-population was characterized by mainly females (78.6% compared with 39.5%, *p* = 0.012), and by using a pump for diabetes management (85.7% compared with 52.7%, *p* = 0.02), as presented in Table 3. Four of them had a report of 3 episodes of DKA, 2 of them had evidence of 2 episodes, and the rest had 1 episode. All patients with recurrent DKA episodes were among patients using a pump.

**Table 3:** Comparison of characteristics of patients living with T1D who had at least one episode of DKA during 6 months prior to combination modality initiation, and those who had not

	Characteristics	DKA episodes	No-DKA episodes	*P* value
Demographic	Sex (Male)	21.4%	60.5%	0.012
	Age (years)	13.7(12.4,17.1)	14.7(12.8,17.3)	0.84
	Age at T1D diagnosis (years)	8.1 (6.5,9.8)	8.3(6.5,10.9)	0.74
	SEP cluster ^	5 (3,6)	7 (4,8)	0.09
	SEP index^	− 0.19(− 0.69,0.42)	0.55(-0.27,1.39)	0.09
Diabetes-related	Pump use	85.7%	52.7%	**0.02**
	Use of CGMS	35.7%	42.1%	0.68
	HbA1c (%)	9.4(8.4,10.6)	9.15(7.9,10.3)	0.39
	HbA1c (mmol/mol)	79(68,92)	77(63,89)	
	Mean glucose	219 (200,276)	230 (181,263)	0.89
	SD of mean glucose levels	90(77,116)	82(73,111)	0.77

After 6 months of using the combination modality, there was a significant decrease in the percent of patients with at least one reported DKA episode from 25.4% to 7.2% (*p* = 0.013). Eleven subjects (20%) experienced at least one episode of DKA at end of follow-up; however, 8 of them were actually not using the combination modality, but switched back to only pump, and one of them experienced again 4 episodes during follow-up. Only 8.8% of those still using the combination modality at end of follow-up had experienced 1 episode of DKA during follow-up. The difference in percent of patients living with T1D and using the combination modality for a median duration of 24.5 months was significantly lower compared to those who switched back to a pump after a median of 16 months (38%), as shown in Table 4. The number of DKA episodes per patient decreased as well from 0.051 at baseline to 0.085 after 6-month follow-up (*p* = 0.002). Only 1 patient had more than 1 episode. At the end of follow-up, 10 patients had 1 episode of DKA, and one reported of 4 episodes. Since follow-up period differed between patients, the rate of DKA episodes per patient month was calculated. It was significantly lower at 6 months and at the last visit 0.020 ± 0.08 and 0.016 ± 0.04 respectively, compared to baseline 0.07 ± 0.15, *P* = 0.011 and *P* = 0.007 respectively), as shown in Table 2. Furthermore, there was again a significant difference in number of episodes per patient month at end of follow-up between those who stayed on the combination modality 0.003 ± 0.009 compared to 0.041 ± 0.07 episodes per patient month among those who returned to conventional modalities (*P* = 0.01).

**Table 4:** Comparison of characteristics between those who stayed on combination modality until end of follow-up and those who switched to MDI or pump only

Characteristics	Patients STILL using combination modality (*n* = 34)	Patients NOT using combination modality (*n* = 21)	*P* value
Characteristics at baseline			
Age at baseline	14.5 (12.7, 18.3)	13.9 (12.3, 16.9)	0.31
Age at diagnosis	8.5 (6.2, 12)	7.5 (6.7, 10)	0.55
Pre-pubertal (Tanner 1–2)	50%	33.3%	0.23
Pubertal (Tanner 3–5)	73.5%	71.4%	
BMI SDS	0.45 (− 0.4, 1.1)	0.71 (− 0.4, 1.0)	0.68
Height SDS	− 0.55 (− 1.3, 0.2)	− 0.53 (− 1.0, − 0.1)	0.81
SEP Cluster	6 (3, 8)	5 (4, 7)	0.51
SEP index	0.42 (− 0.4, 1.3)	− 0.001 (− 0.3, 0.6)	0.47
Mean Glucose (mg/dL)	219 (178, 257)	235 (190, 269)	0.43
SD of mean glucose levels	90 (76.5,111.5)	81 (74,111)	0.56
HbA1c %	9.25 (8.20, 10.07)	9.1 (8.2, 10.4)	0.82
HbA1c mmol/mol	78 (66, 87)	76 (66, 90)	
Use of CGMS	38.2%	33.3%	0.99
% time spent in 70–180 mg/dL	47 (35,7.5)	40 (26,47.5)	0.46
% time spent in > 180 mg/dL	52 (31,64)	59 (48,73)	0.27
% time spent in < 70 mg/dL	1 (0.78,12.5)	1 (0,13)	0.28
Insulin total daily dose U/kg/d	0.85 (0.67,0.95)	0.96 (0.72,1.0)	0.46
% Basal insulin/d	47.0 (37,60)	50 (39,54)	0.94
% of patients with DKA history	20.5%	33.3%	0.30
DKA episodes per patient^a^	0.37 ± 0.83	0.60 ± − 0.99	0.31
DKA episodes per patient month^a^	0.06 ± 0.13	0.09 ± 0.16	0.30
Characteristics at end of follow-up			
Duration of combination modality (months)	24.5 (14.25, 52.25)	16 (10, 27)	**0.009**
Mean Glucose (mg/dL)	201 (167,252)	200 (183,250)	0.67
SD of mean glucose levels	77 (67.5,98)	96 (65,115)	0.68
HbA1c %	9.7 (7.9,10.6)	9 (8.1,10.6)	0.95
HbA1c mmol/mol	82.5 (62.8,92.4)	74.9 (65,92.4)	
Use of CGMS	61.7%	50%	0.13
% time spent in 70–180 mg/dL	55 (24.5,68.5)	53 (33.6,67.7)	0.95
% time spent in > 180 mg/dL	38 (23.7,67.7)	42 (36,47)	0.90
% time spent in < 70 mg/dL	4 (1,13)	2 (0.5,13)	0.90
Insulin total daily dose U/kg/d	0.88 (0.72,1.14)	1.03 (0.87,1.36)	0.13
% Basal insulin/d	46.3 (39.5,57)	42 (35,50)	0.28
% patients with DKA during follow-up	8.8%	38.1%	**0.02**
DKA episodes per patient^a^	0.10 ± 0.30	0.55 ± 0.94	**0.01**
DKA episodes per patient month^a^	0.003 ± 0.09	0.04 ± 0.07	0.01

The socioeconomic position (SEP) by home address was analyzed based on the Israel Central Bureau of Statistics’ Characterization and Classification of Statistical Areas within Municipalities and Local Councils by the Socio-Economic Level of the Population 2015 [21], DKA, diabetes ketoacidosis

^a^Presented as mean and standard deviation. Bold indicates significant

### Glycemic parameters during follow-up

Most glycemic control parameters, including HbA1c, percent of time spent in, above, and below range, TDDi, and frequency of severe hypoglycemic events, did not change significantly during the first 6 months after initiation of the combination modality and at the end of follow-up compared to the baseline values, as shown in Table 2. Mean glucose and its standard deviation score improved during the first 6 months after initiation of combination modality, but improvement was not sustained, and its clinical significance is probably negligible. Glycemic control data of times in range were available at baseline for only 21 adolescents (38%) of the population using CMGS, at 6 months for only 22 adolescents (40%), and 28 (50%) at end of follow-up. Although there was a significant increase in the use of CGMS during follow-up, it was not large enough to show significant change.

### Subgroup analysis at end of follow-up

As reported, there was a significant difference in DKA outcome among those who stayed compared to those who stopped the modality. At the end of follow-up, 34 (61.8%) study participants were still using the combination modality, while 21 (38.2%) had switched to a conventional modality (pump *n* = 12, 21.8% and MDI *n* = 9, 16.4%). There were no significant predictive differences at baseline demographic, clinical, and glycemic characteristics between those who remained on the combination modality and those who switched back to conventional modalities, and the only long-term difference was in frequency of DKA episodes, and not in glycemic control parameters, as demonstrated in Table 4.

## Discussion

We describe a unique cohort of children and youth with T1D who used a combination modality of short-acting insulin delivered by pump for boluses only and full or partial basal replacement by subcutaneously injected long-acting insulin once daily. This approach evolved due to the need to help poorly controlled people living with T1D, who wish to use pump therapy for quality of life, and still be safe. This modality enabled them to have the benefits of spontaneity, flexibility and avoiding the need of several daily injections, while decreasing the risk of DKA.

Adolescents with poorly controlled T1D whose insulin is delivered by pumps are reportedly at increased risk for DKA episodes [10, 11], although followed by multi-disciplinary teams. Additionally, some people living with diabetes may find it distressing and inconvenient to be continuously attached to a pump, particularly during sports, water activities, sleep, and sexual intercourse. These issues were the main reported medical team and caregivers’ reasons for switching to the combination modality in our young cohort. Those challenges have been previously addressed, and several case reports described the use of supplemental subcutaneous long-acting insulin instead of the pump basal infusion. For example, Phillips et al. [17] described a 56-year-old patient who was experiencing recurrent episodes of DKA and who replaced 60% of the basal insulin with injected insulin glargine, resulting in the cessation of the DKA episodes. Aronson et al. [18] demonstrated a high safety profile of combined therapy with pump and injected insulin Degludec (50% of the basal dose) in adults with T1D who removed their pump before extended periods of exercise. Johansson et al. [20] demonstrated that partial replacement with glargine during pump treatment with Lispro protected against the development of ketosis in a group of seven adults with T1D. Alemzadeh et al. [19] reported the safety of partial basal insulin replacement with glargine in a group of 12 children with T1D treated with insulin pumps. These reports, however, involved small numbers of participants and with partial basal insulin replacement, and only one study was conducted in a pediatric population. [19].

To the best of our knowledge, our study represents the most comprehensive analysis to date of the practical use of that combined modality, including both partial and full replacement of basal insulin with injected long-acting insulin in young individuals. Our findings underscore the significance of this treatment option, even in the era of the powerful tool of AHCL systems, since a large percentage of the world population living with T1D still uses the older management fashions due to availability and financial causes. Notably, most of the indications for initiating the combined modality in our study population align with individuals who may not fit to the AHCL option, or may not agree to use it, further emphasizing its importance.

The size of our study cohort enabled us to characterize the population most likely to need that alternative modality, especially the poorly controlled adolescents, who were pubertal on physical examination (71%), and possibly those who had been diagnosed with ADHD. Pubertal stage was reported as being a risk factor for poorer outcome, even when treated with AHCL [26, 27]. ADHD, is also a known risk factor for lack of compliance, recurrent DKA episodes, and poorer glycemic control [25, 26]. However, the diagnosis of ADHD should be regarded with caution, since it had been suggested that there may be over diagnosis of ADHD worldwide [30], and even more in Israel. [31]. The prevalence of ADHD is variable, affecting 2–16% of the school aged population [28], and evidence exists that there is a 1.5 higher risk of having ADHD among people living with T1D compared with their siblings [29]; in our cohort, it was reported among 20% of participants.

As expected, glycemic control did not change with the combination modality, since the administration of insulin is not the only parameter responsible for glycemic control. It should be noted that most patients in this cohort were the more challenging population to balance (mean HbA1c of above 9%, and lower SES). The study emphasizes once again how difficult it is to improve diabetic control, and that just changing insulin administration modality is not sufficient. However, changing insulin delivery modality may change the risk for DKA episodes among the poorly controlled population with T1D.

The strength of our study lies in its novelty in describing and proving the benefit of a modality used by physicians worldwide, as an off-label option, with no evidence so far of its assumed benefit. Our study findings shed light on subjects’ characteristics, who may benefit from this modality, mainly by significantly reducing DKA episodes, while still enjoying the benefits of a pump for boluses.

There are some limitations to our study that bear mention, primarily, its retrospective design. Due to the real-life retrospective design, the protocol for the initiation of a combination modality and its education paradigm were not uniform, but rather determined and explained by the subject’s multi-disciplinary team, and the reasons for discontinuing the combined therapy are missing. Additionally, our study population is small, although nationwide, since we use this modality only as last option and for a very specific population of patients. Therefore, it was possibly not large enough to detect baseline characteristics differences among those who developed DKA episodes and those who did not, and among those who stopped the combination modality.

## Conclusion

The findings of our study suggest that although the most physiological modality nowadays is AHCL, until it is available to all, and accepted to be used by all, sometimes “out of the box” solutions for specific challenging patients should be suggested. The combination modality of rapid-acting insulin delivered by pump for boluses in combination with subcutaneously injected long-acting insulin once daily may enable all benefits of a pump use among youth with T1D, without deleteriously affecting glycemic control, and while providing protection from DKA. This treatment strategy is mainly suitable for the pubertal ones, with poorly controlled diabetes. It may also be welcomed by young people living with diabetes who reject constant pump use due to body image or intensive physical activity, but still want the benefit of delivering insulin without an additional subcutaneous injection.

## Data Availability

Anonymized data that express the results reported in this article can be made available upon reasonable request to the corresponding author and will require the completion of a data processing agreement.
